# CUL3 and COPS5 Related to the Ubiquitin-Proteasome Pathway Are Potential Genes for Muscle Atrophy in Mice

**DOI:** 10.1155/2022/1488905

**Published:** 2022-06-30

**Authors:** Qun Xu, Jinyou Li, Ji Yang, Zherong Xu

**Affiliations:** Department of Geriatrics, The First Affiliated Hospital, Zhejiang University, School of Medicine, No. 79, Qingchun Road, Hangzhou, Zhejiang, China

## Abstract

Sarcopenia is a condition that reduces muscle mass and exercise capacity. Muscle atrophy is a common manifestation of sarcopenia and can increase morbidity and mortality in specific patient populations. The aim of this study was to identify novel prognostic biomarkers for muscle atrophy and associated pathway analysis using bioinformatics methods. The samples were first divided into different age groups and different muscle type groups, respectively, and each of these samples was analyzed for differences to obtain two groups of differentially expressed genes (DEGs). The two groups of DEGs were intersected using Venn diagrams to obtain 1,630 overlapping genes, and enrichment analysis was performed to observe the Gene Ontology (GO) functional terms of overlapping genes and the enrichment of the Kyoto Encyclopedia of Genes and Genomes (KEGG) pathway. Subsequently, WGCNA (weighted gene coexpression network analysis) was used to find gene modules associated with both the age and muscle type to obtain the lightgreen module. The genes in the key modules were analyzed using PPI, and the top five genes were obtained using the MCC (maximum correntropy criterion) algorithm. Finally, CUL3 and COPS5 were obtained by comparing gene expression levels and analyzing the respective KEGG pathways using gene set enrichment analysis (GSEA). In conclusion, we identified that CUL3 and COPS5 may be novel prognostic biomarkers in muscle atrophy based on bioinformatics analysis. CUL3 and COPS5 are associated with the ubiquitin-proteasome pathway.

## 1. Introduction

The skeletal muscle consists of muscle fibers and bundles that play an important role in regulating the metabolic aspects of the body. Universally, increased exercise leads to increased muscle mass, while decreased or restricted rates of exercise can lead to muscle atrophy [1]. Muscle atrophy is a response that can weaken patients with hunger and some systemic diseases, often leading to muscle mass loss [2], and this phenomenon is also defined as sarcopenia in a broad sense. Muscular dystrophy is usually manifested as generalized muscular atrophy and fat infiltration, which is associated with the incidence rate and mortality in the context of aging and cancer [3]. For muscle atrophy, when muscles are inactive for a long period of time, muscle contractile function and muscle fiber size decrease as a result of increased degradation and decreased synthesis of muscle proteins [4]. Muscle atrophy can occur in a variety of individuals who are suffering from diseases, such as diabetes, cancer, muscle genetic disorders, and neurodegenerative diseases, or under mechanical unloading conditions, such as prolonged bed rest and reduced step count [5]. Muscle atrophy can lead to a poorer functional status, reducing the quality of life of patients and increasing morbidity and mortality in specific patient groups [6].

Muscle atrophy is the direct manifestation of sarcopenia, and its main cause is excessive protein degradation. The Forkhead box O (FOXO) family of transcription factors and the progrowth IGF-AKT pathway are key mediators of muscle atrophy and are important for the protein degradation pathway in muscle atrophy [7]. Also, mitochondrial dysfunction is associated with disuse muscle atrophy [8]. Mitochondrial oxidative stress (OS) can stimulate muscle proteolysis by increasing the involvement in protein degradation pathways and protein expression [9], and excessive oxidative stress can lead to muscle damage and, consequently, muscle atrophy [10]. The occurrence of muscle atrophy is both “active” and “passive”; therefore, its study is of high clinical necessity. “Active” muscle atrophy is often caused by denervation. Nutritional factors that maintain skeletal muscle function require the innervation of motor neurons, and when muscle innervation deteriorates, it can lead to inadequate nerve input, resulting in muscle weakness or even atrophy [11]. This loss of muscle mass and function caused by peripheral nervous system injury or motor neuron disease is called neurogenic muscle atrophy and, in some cases, can reduce survival rates [12], which is also considered to be the main cause of the progression of sarcopenia. “Passive” muscle atrophy, on the other hand, is usually the result of other conditions that require reduced or limited activity. It is called disuse muscle atrophy and promotes skeletal muscle atrophy by stimulating protein decomposition [13]. Generally speaking, with the progress of aging, reactive oxygen species produced by muscle mitochondria will increase [14], which is another reason to promote the occurrence of sarcopenia. In contrast, muscle atrophy associated with wasting after a musculoskeletal injury is often difficult to overcome and can persist despite efforts at rehabilitation [15].

Several studies have shown that the development of muscle atrophy is associated with the regulation of proteins or RNAs. Li et al. [6] showed that miR-29b causes several types of muscle atrophy, while Li et al. demonstrated that lncIRS1 indirectly controls the production of muscle atrophy [16]. In this study, we will use bioinformatics approaches to find potential genes associated with muscle atrophy by grouping mice and obtaining sequencing information.

## 2. Materials and Methods

### 2.1. Establishment of the Animal Model

In this study, a total of 30 6-week-old male mice were selected from Institute of Cancer Research (ICR) (Cavens Lab, China) and divided into different age groups and muscle types groups. Firstly, 30 mice were reared at 25°C in light/dark cycle environment for 12 h each, without restriction of movement and feeding. At 2 months, 15 mice were sacrificed by neck removal, extensor digitorum longus atrophy(EDLA) tissues were obtained from 7 mice, and soleus longus atrophy (SOLA) tissues were obtained from 8 mice, the remaining 15 mice were sacrificed by neck removal at 29 months, and EDLA tissues were obtained from 7 mice. SOLA tissues were obtained from 8 mice. Finally, 30 muscle atrophy samples were obtained, including 7 2-month-old EDLA muscle atrophy samples, 8 2-month-old SOLA muscle atrophy samples, 8 29-month-old EDLA muscle atrophy samples, and 7 29-month-old SOLA muscle atrophy samples. The samples were sent for sequencing to obtain the muscle atrophy-related gene expression profile. Animal experiments were carried out in the First Affiliated Hospital of Zhejiang University, and the experimental program was carried out in an accredited animal facility (no. 2017-038).

### 2.2. Differential Expression Analysis

Samples were grouped according to the monthly age or muscle type, and two groups of differentially expressed genes (DEGs) were obtained using the R package and the limma package for differential analysis of two-month-old and 29-month-old mice or EDLA and SOLA muscle atrophy mice, respectively. Genes with differential expression in the two groups were selected using |log2FC| >1, *P* < 0.05, as a screening condition. The overlapping genes were obtained by taking the intersection of the two groups of DEGs using a Venn diagram.

### 2.3. Weighted Gene Coexpression Network Analysis (WGCNA)

WGCNA was conducted using the R package to analyze the co-expression of DEGs associated with muscle atrophy in mice. A soft threshold *β* was calculated using the scale-free topology criterion to generate a weighted adjacency matrix. Subsequently, gene modules were cut using the dynamic tree-cutting method. Additionally, the correlation between each gene module and sample phenotype was analyzed by the WGCNA package, and the module that correlated the most with both the monthly age and muscle type was selected as the target gene module.

### 2.4. Protein-Protein Interaction (PPI) Network

The Search Tool for the Retrieval of Interacting Genes/Proteins (STRING) database was used to explore the known and predicted interactions between proteins, including direct and indirect relationships, construct protein expression networks in key gene modules from the STRING database, and link disease-causing gene-to-gene features via Cytoscape. The top five extracted genes were processed using the maximum correntropy criterion (MCC) algorithm in the cytoHubba plugin and used as pivotal genes.

### 2.5. Gene Expression Validation

To understand the differences in the expression of each pivotal gene between different phenotypes in mice, the expression level distribution was achieved using the R package ggplot2. The samples were grouped according to monthly age and muscle type, and *t*-tests were used to analyze the significance of differences in pivotal gene expression. *P* < 0.05 was considered statistically significant.

### 2.6. Enrichment Analysis

To better understand the pathogenic role of mRNA, overlapping genes were analyzed for Gene Ontology (GO) function and Kyoto Encyclopedia of Genes (KEGG) pathways using the R package ClusterProfiler. GO is used to annotate genes with function, and it contains a molecular function (MF), biological process (BP), and cellular component (CC). In contrast, KEGG can be used to analyze the gene functions and functional information of the related genomes and to explore the functional pathways of pathogenic genes. In the enrichment results, *P* < 0.05 or FDR < 0.05 was considered significant for the enriched pathway. Gene set enrichment analysis (GSEA) was used to analyze the association of hub genes with the mouse muscle atrophy pathway. KEGG pathways in the top three of significance were selected for analysis using the screening conditions of |NES| >1, NOM *p* val <0.05, and FDR *q* val <0.25.

## 3. Analysis of Results

### 3.1. Differential Expression Analysis

**Figure 1:**
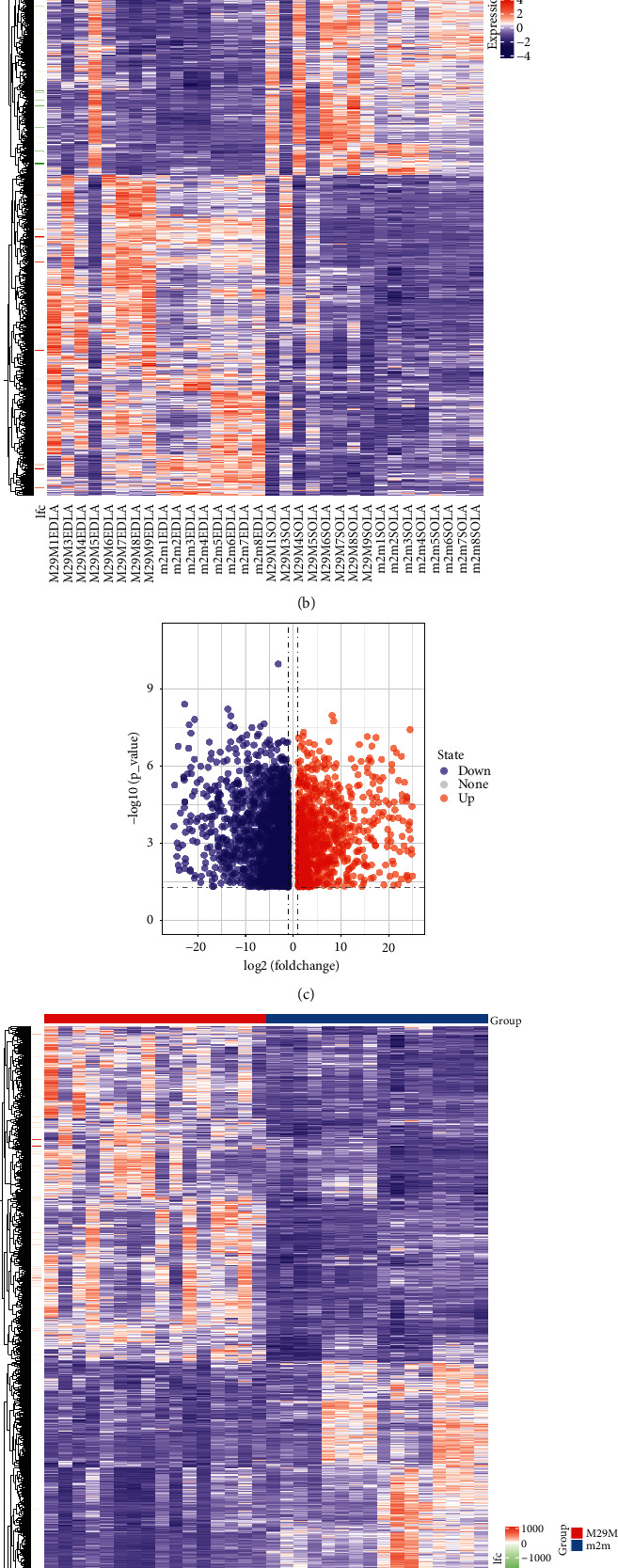
Results of differential gene expression analysis in different group. (a, b) Differential gene expression results after grouping based on different muscle types. (c, d) Differential gene expression results after grouping based on different monthly ages of mice. (e) DEGs obtained based on monthly age grouping are named months, and DEGs obtained based on muscle type grouping are named muscle. The intersection of the two groups of DEGs was obtained using the Venn diagram.

The mice were divided into two groups according to their age and the type of muscle atrophy, and each group was analyzed for differences. A total of 2,721 genes were downregulated, and 1,298 genes were upregulated in the two-month-old and 29-month-old groups of muscle atrophy mice (Figures 1(a) and 1(b)). In the EDLA muscle atrophy group and the SOLA muscle atrophy group, a total of 2,651 genes were downregulated and 1,148 genes were upregulated (Figures 1(c) and 1(d)). For the potential causative genes of muscle atrophy in mice, this study used Venn diagrams to take the intersection of the months and muscle groups, and obtained 1,630 overlapping genes associated with both the monthly age and muscle type (Figure 1(e)).

### 3.2. GO Function and the KEGG Pathway

**Figure 2:**
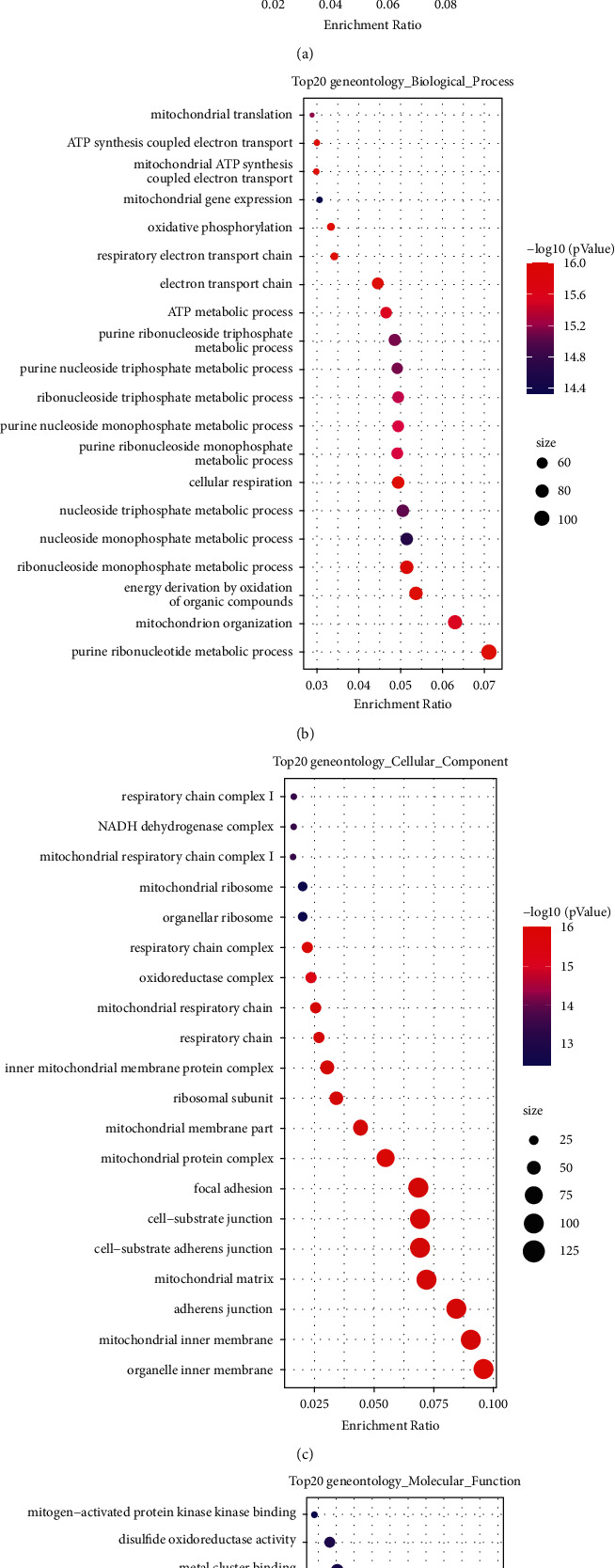
Results of enrichment analysis of DEGs. (a) Results of the top 20 KEGG pathways. (b–d) Results of the top 20 BP, CC, and MF functions, respectively.

To understand the function of overlapping genes in muscle atrophy in mice, we used enrichment analysis to observe the main functions and pathways of these genes. The top 20 KEGG pathways and GO function terms were enriched and presented using bubble plots. The results showed that the target genes were associated with KEGG processes, such as Parkinson's disease, Alzheimer's disease, and oxidative phosphorylation (Figure 2(a)). For GO functional terms, the target genes were significantly enriched for energy metabolic processes, such as purine nucleotide metabolic processes; energy production from oxidation of organic compounds; ribonucleoside monophosphate metabolic processes (Figure 2(b)); cellular components, such as organelle inner membrane; mitochondrial inner membrane; mitochondrial protein complexes (Figure 2(c)); and molecular functions, such as cell adhesion molecule binding, electron transfer activity, and NADH dehydrogenase activity (Figure 2(d)).

### 3.3. WGCNA Selection of Key Gene Modules

**Figure 3:**
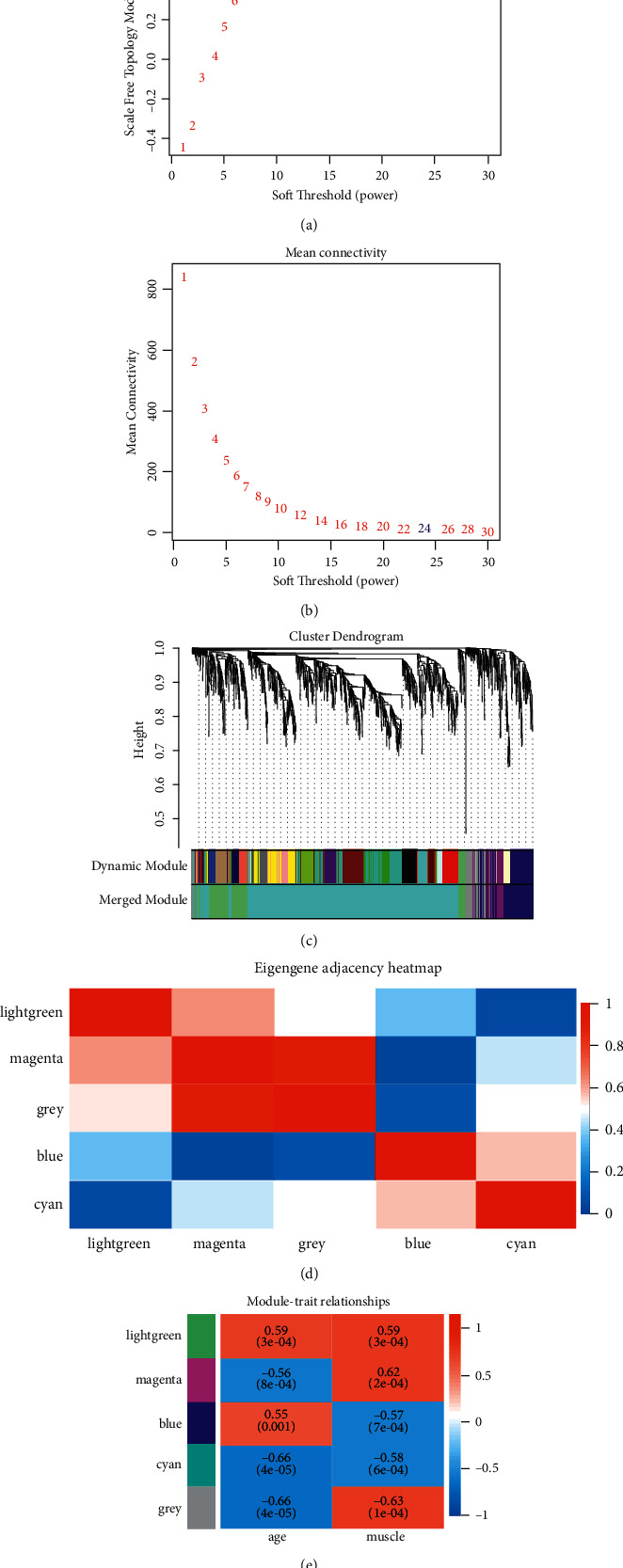
Weighted mouse muscle atrophy gene coexpression. (a, b) T scale-free fit indices and mean connectivity for various soft threshold powers. (c) Dendrogram of overlapping genes based on different index clusters. (d) Neighbor-joining heat map of modular signature genes. (e) Correlation heat map of modular signature genes with different phenotypes of muscle atrophy in mice.

In WGCNA, when the correlation coefficient is greater than 0.85, the optimal soft threshold value *β* = 24 (Figures 3(a) and 3(b)). At this time, the genes can be clustered using the average chain hierarchy clustering method, and five color modules can be obtained (Figure 3(c)). The lightgreen, cyan, blue, grey, and magenta modules contain 224, 1074, 175, 93, and 64 genes, respectively (Figure 3(d)). Among them, the lightgreen module was significantly and positively correlated with both mouse month age (cor = 0.59, *P*=0.0003) and muscle type (cor = 0.6, *P*=0.0003).

### 3.4. Protein Interaction Network to Find Pivotal Genes

**Figure 4:**
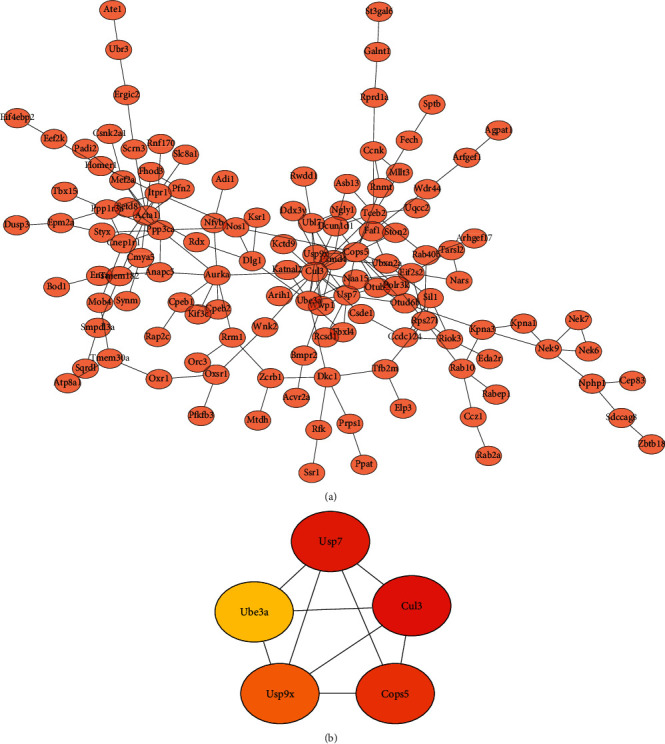
PPI results. (a) Map of protein interaction network relationships in the lightgreen module. (b) Top five significant genes.

To further identify pivotal genes affecting the progression of muscle atrophy in mice, we used the STRING database to identify the interactions between proteins in the lightgreen module and obtained a total of 223 nodes and 196 edges (Figure 4(a)). The top five genes in terms of correlation ranking such as ubiquitin-specific peptidase 7(USP7), ubiquitin-protein ligase E3A (UBE3A), Cullin 3 (CUL3), ubiquitin-specific peptidase 9, X-linked (USP9X), and COP9 constitutive photomorphogenic homolog subunit 5 (COPS5) were then derived using the MCC algorithm and used as pivotal genes for subsequent analysis (Figure 4(b)).

### 3.5. CUL3 and COPS5 as Pivotal Genes

**Figure 5:**
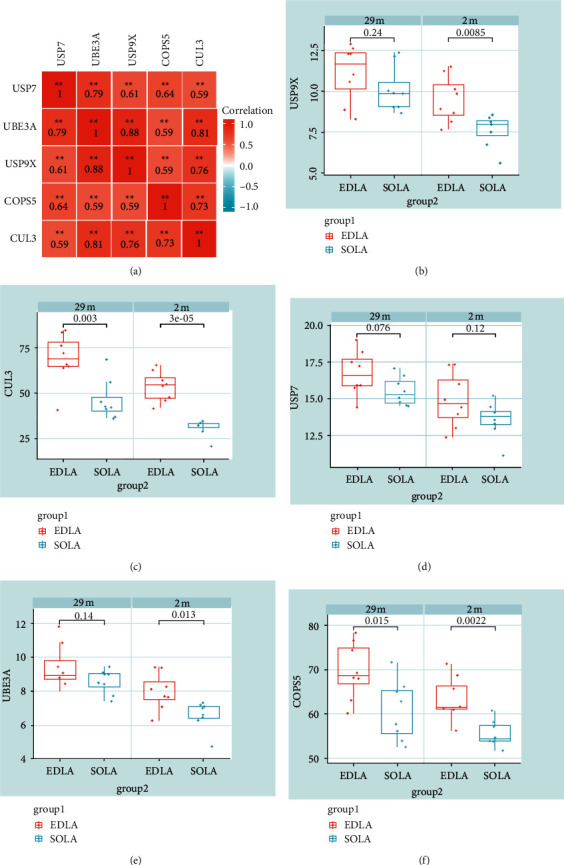
Expression level distribution of pivotal genes. (a) Correlation analysis of expression levels of five pivotal genes, ^*∗*^*P* < 0.05 and ^*∗∗*^*P* < 0.01. (b–f) The expression levels of USP9X, CUL3, USP7, UBE3A, and COPS5 in different muscle type groups were observed by monthly age grouping, respectively.

Correlation analysis of pivotal genes revealed high correlation coefficients among all five genes and significant results (Figure 5(a)). The expression levels of the five genes were then analyzed, and the mouse samples were divided into 29-month-old and two-month-old to observe the difference in the expression of pivotal genes in the EDLA muscle and the SOLA muscle, respectively. The results showed that only the expression levels of CUL3 and COPS5 were significant at the same time (Figures 5(b)–5(f)), which indicated that CUL3 and COPS5 were correlated with both months of age and muscle type during the development of muscle atrophy in mice.

### 3.6. GSEA Analysis of the KEGG Pathway of Key Genes

**Figure 6:**
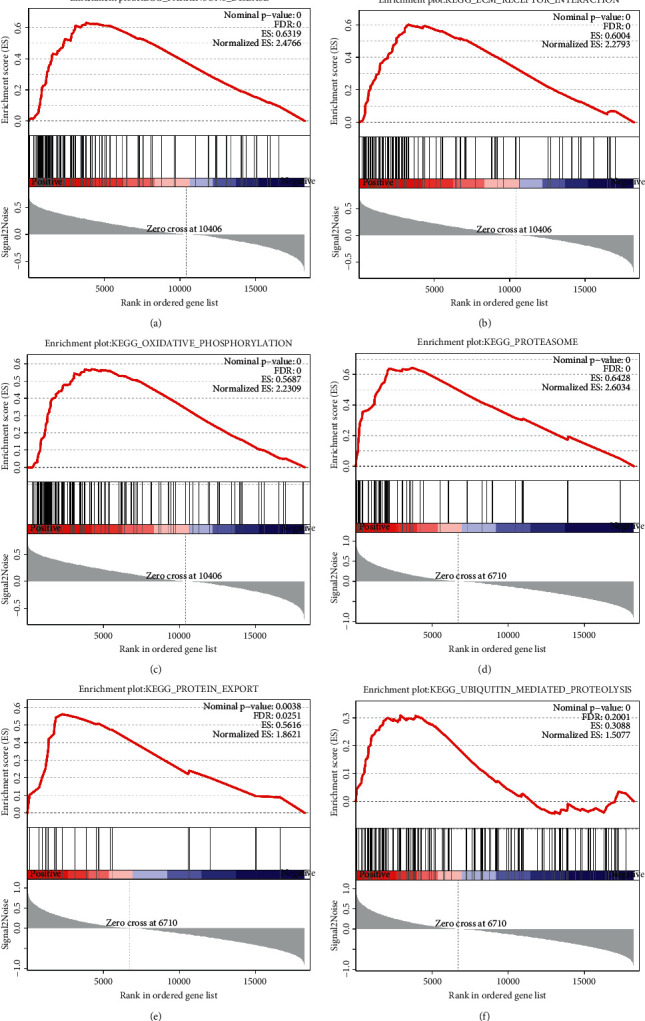
GSEA results of key genes. (a–c) GSEA results of the top three ranked CUL3 in mouse muscle atrophy. (d–f) GSEA results of the top three ranked COPS5 in mouse muscle atrophy.

To further understand the functional pathways involved in the key genes in mouse muscle atrophy, this study focused on the KEGG pathway using GSEA. Analysis showed that CUL3 was associated with signaling pathways, such as Parkinson's disease, ECM-receptor interaction, and oxidative phosphorylation (Figures 6(a)–6(c)), while COPS5 was associated with signaling pathways, such as the proteasome, protein export, and ubiquitin-mediated protein hydrolysis (Figures 6(d)–6(f)).

## 4. Discussion

Muscle reduction is the main cause of muscle strength and muscle mass loss in the elderly. It is also an important complication of chronic kidney disease [17], cardiac cachexia [18], diabetes mellitus [19], and cancer [20]. Factors associated with sarcopenia include neurological factors, oxidative stress, mitochondrial dysfunction, fat accumulation, mild inflammation, nutritional deficiency, hormone changes, reduction of the number of microcells, and regenerative capacity. Some studies have shown that moderate exercise enhances mitochondrial adaptation and function, which facilitates skeletal muscle recovery and myocyte regeneration [21]. In addition, many types of drugs have the potential to treat muscle atrophy, including anabolic drugs, enzyme inhibitors, and anti-inflammatory drugs [22]. But so far, no drugs have been approved for the treatment of sarcopenia, which makes the search for appropriate intervention targets become the focus of clinical research. Advances in transcriptome technology and high-throughput analysis allow the detection of a variety of cytokines, such as growth factors, transcription factors, cell signal pathway activators, and noncoding RNA, which are involved in the regulation of gene expression during skeletal muscle aging changes and adaptation [23]. In this study, an expression matrix containing 18234 genes was obtained by sequencing mouse samples. The differential expression of these genes was compared from two dimensions, and 1630 overlapping genes were obtained.

Further enrichment analysis was used to clarify the role of 1630 DEGs. KEGG enrichment analysis showed that the differential genes were related to Parkinson's disease, Alzheimer's disease, and oxidative phosphorylation. Parkinson's disease (PD) is a progressive dyskinesia. Sarcopenia is common in patients with Parkinson's disease and is associated with more advanced disease stages, higher dyskinesia and nonexercise load, falls, reduced quality of life, and hospitalization. Sarcopenia and Parkinson's disease have multiple common pathways, which may affect each other's prognosis and patients' quality of life [24]. In addition, GO function analysis showed that mitochondrial changes were also an important pathway of muscle atrophy. Previous studies have shown that mitochondrial biosynthesis, mitochondrial respiratory complex subunit expression, mitochondrial respiration, and ATP levels are significantly reduced in aging skeletal muscle [25, 26]. Migliavacca et al. demonstrated for the first time that mitochondrial biological dysfunction is the strongest molecular feature of sarcopenia. Changes in mitochondria include decreased mitochondria, decreased expression and activity of mitochondrial respiratory complexes, and interference with NAD biosynthesis and repair in muscle [27].

In order to further study the gene regulatory factors related to sarcopenia, five key genes were identified by WGCNA and PPI. The expression of CUL3 and COPS5 was significantly correlated with mouse month age and muscle type. CUL3 (Cullin 3) is a key protein of the E3 ubiquitin ligase complex and mediates the proteasomal degradation process [28]. CUL3 regulates a variety of cellular functions, such as antioxidant, cell cycle, protein transport, and signal transduction [29]. CUL3 deficiency increases the expression of cell cycle proteins E and P21, which are associated with abnormal proliferation, DNA damage, and apoptosis [30]. Studies have shown that CUL3 is associated with vascular smooth muscle specificity and may indirectly promote proliferation, migration, and inflammatory response of vascular smooth muscle cells [31]. In contrast, its deletion promotes NO reactivity and leads to atherosclerosis and hypertension [32]. In addition, CUL3 plays a key role in the development and function of the transverse muscle, and it mediates protein homeostasis, with important implications in muscle function [33]. Additional studies have used gene expression and structural analysis to identify CUL3 associated with sarcopenia [34]. Another key gene is COP9 signaling vesicle complex 5 (COPS5), also known as C-Jun activation domain-binding protein-1 (Jab1), a multifunctional protein [35]. COPS5 is involved in the regulation of cellular and developmental processes, such as signal transduction, cell cycle processes, DNA damage response, and tumorigenesis [36], and plays an important role in ubiquitin-mediated protein degradation [37]. Most of the current studies focus on the oncogenic function of COPS5, and its dysregulated activity contributes to the development of cancers, such as breast cancer [38], glioma [39], and prostate cancer [40]. It has been reported that COPS5 usually interacts with proteins or binds to miRNAs to regulate tumor progression [41], and it has also been shown to affect the metastatic potential of cancer cells by inhibiting SNAIL ubiquitination [42]. Regarding the role in muscle function, the results of Velardo et al. showed that COPS5 has an important role in muscle development, maintenance, and regeneration and is associated with the pathogenesis of congenital muscular dystrophy [43].

In order to further understand the functional pathway of the two genes involved in mouse muscle atrophy, CUL3 and COPS5 were analyzed by GSEA. GSEA results showed that CUL3 was related to Parkinson's disease, ECM-receptor interaction, and oxidative phosphorylation. COPS5 is related to the proteasome, protein output, and ubiquitin-mediated proteolysis. Previous studies have shown that the ubiquitin-proteasome system (UPS) is activated during senile muscle atrophy [44]. CUL3 [29] and COPS5 [45] are important members of the ubiquitin-proteasome system, which is consistent with our study. The ubiquitin-proteasome pathway is the main protein degradation pathway, and the process is a three-step enzymatic cascade reaction [46]. Furthermore, the E3 ligase of the Cullin family catalyzes the last step, which attaches ubiquitin protein to the target substrate protein [47]. Interestingly, Blondelle et al. in their study showed that the regulation of the Cullin activity is associated with the COP9 signaling vesicle complex [48]. This study is similar to the results of Spearman analysis in the present study, where CUL3 was correlated with COPS5 (cor = 0.73) (Figure 5(a)).

## 5. Conclusion

In conclusion, this study identified two new potential genes (CUL3 and COPS5) of muscle atrophy. Furthermore, CUL3 and COPS5 are related to the ubiquitin-proteasome pathway, which may provide useful ideas for the study of the pathogenesis of muscle atrophy. In the future, we will continue to study in vitro and in vivo.

## Data Availability

All data generated or analyzed during this study are included in this article.
